# Artificial Intelligence–Enabled Software Prototype to Inform Opioid Pharmacovigilance From Electronic Health Records: Development and Usability Study

**DOI:** 10.2196/45000

**Published:** 2023-07-18

**Authors:** Alfred Sorbello, Syed Arefinul Haque, Rashedul Hasan, Richard Jermyn, Ahmad Hussein, Alex Vega, Krzysztof Zembrzuski, Anna Ripple, Mitra Ahadpour

**Affiliations:** 1 Center for Drug Evaluation and Research US Food and Drug Administration Silver Spring, MD United States; 2 Neuromuscular Institute Rowan-Virtua School of Osteopathic Medicine Stratford, NJ United States; 3 Lister Hill National Center for Biomedical Communications National Library of Medicine–National Institutes of Health Rockville, MD United States

**Keywords:** electronic health records, pharmacovigilance, artificial intelligence, real world data, EHR, natural language, software application, drug, Food and Drug Administration, deep learning

## Abstract

**Background:**

The use of patient health and treatment information captured in structured and unstructured formats in computerized electronic health record (EHR) repositories could potentially augment the detection of safety signals for drug products regulated by the US Food and Drug Administration (FDA). Natural language processing and other artificial intelligence (AI) techniques provide novel methodologies that could be leveraged to extract clinically useful information from EHR resources.

**Objective:**

Our aim is to develop a novel AI-enabled software prototype to identify adverse drug event (ADE) safety signals from free-text discharge summaries in EHRs to enhance opioid drug safety and research activities at the FDA.

**Methods:**

We developed a prototype for web-based software that leverages keyword and trigger-phrase searching with rule-based algorithms and deep learning to extract candidate ADEs for specific opioid drugs from discharge summaries in the Medical Information Mart for Intensive Care III (MIMIC III) database. The prototype uses MedSpacy components to identify relevant sections of discharge summaries and a pretrained natural language processing (NLP) model, Spark NLP for Healthcare, for named entity recognition. Fifteen FDA staff members provided feedback on the prototype’s features and functionalities.

**Results:**

Using the prototype, we were able to identify known, labeled, opioid-related adverse drug reactions from text in EHRs. The AI-enabled model achieved accuracy, recall, precision, and *F*_1_-scores of 0.66, 0.69, 0.64, and 0.67, respectively. FDA participants assessed the prototype as highly desirable in user satisfaction, visualizations, and in the potential to support drug safety signal detection for opioid drugs from EHR data while saving time and manual effort. Actionable design recommendations included (1) enlarging the tabs and visualizations; (2) enabling more flexibility and customizations to fit end users’ individual needs; (3) providing additional instructional resources; (4) adding multiple graph export functionality; and (5) adding project summaries.

**Conclusions:**

The novel prototype uses innovative AI-based techniques to automate searching for, extracting, and analyzing clinically useful information captured in unstructured text in EHRs. It increases efficiency in harnessing real-world data for opioid drug safety and increases the usability of the data to support regulatory review while decreasing the manual research burden.

## Introduction

Postmarketing drug safety surveillance at the Center for Drug Evaluation and Research (CDER) of the US Food and Drug Administration (FDA) aims to detect, characterize, monitor, and prevent adverse drug reactions (ADRs) for FDA-approved drugs and therapeutic biologic products. Biomedical resources used to detect adverse drug event (ADE) safety signals include clinical trials, spontaneous adverse event (AE) reports submitted to the FDA Adverse Events Reporting System (FAERS), published scientific reports in the literature, and others. The FAERS database compiles AE and medication error reports submitted to the FDA to support postmarket drug safety monitoring. FAERS monitoring has yielded information on rare ADEs, but the information is limited by underreporting [1,2]. Multimodal approaches to pharmacovigilance using multiple biomedical resources may offer improved drug safety signal detection compared to reliance on single resources [3].

Electronic health records (EHRs) are a rich source of real-world information that may potentially serve as a new complementary drug safety resource. Although not specifically created to document ADEs, the EHR may provide information about product side effects, including those that occur a prolonged time following initial drug exposure [4], and may contribute to assessments of the safety of generic and pediatric drug products [5,6]. EHRs have been explored to complement ADE signal identification from spontaneous AE reports [7].

Published scientific reports describe various natural language processing (NLP) and artificial intelligence (AI)-based approaches to analyzing text from EHRs for ADE detection and pharmacovigilance. Named entity recognition (NER) to identify drug and AE mentions in text followed by extraction of the relationships between those entities is a critical technical challenge in building successful analytical algorithms. In general, keywords, rule-based algorithms, and machine learning methods have been used for case detection [8]. Some early studies used trigger phrases to screen the text of discharge summaries for AE concepts [9,10]. Established NLP algorithms applied to AE detection include MedLEE, which identifies clinical concepts and cross-maps them to Unified Medical Language System (UMLS) concepts [11]; MetaMap, which processes biomedical text and maps it to the UMLS [12]; and Clinical Text Analysis and Knowledge Extraction System (cTAKES), an NLP system that incorporates rules and machine learning [13]. More recent studies use multiple NLP models, including long short-term memory (LSTM), conditional random field (CRF), support vector machines (SVMs), and bidirectional encoder representations from transformers (BERTs) [14]. Shared task challenges designed to promote advances in NLP for drug safety and ADE detection from EHRs have been conducted in recent years, including the MADE 1.0 challenge [15] and the n2c2 Clinical Challenge [16]. Text analytic engines, such as Amazon Comprehend Medical, Microsoft Text Analytics for Health, and the Google Healthcare Natural Language application programming interface, are deep learning–based pretrained models. These models can perform a variety of general health care NLP tasks, such as NER, relation detection, entity disambiguation, and others [17]. We combine a similar deep learning model with domain-specific, rule-based algorithms from domain expertise to detect opioid-related ADEs (ORADEs) from clinical notes.

Using novel AI methods, time-consuming manual chart review can be automated to provide active surveillance with enhanced detection of emerging product safety issues in near–real time. Opioids are one of the most frequently implicated drug classes for ADRs in hospitalized patients and are associated with confusion, constipation, respiratory depression, sedation, ileus, hypotension, and other ADRs [18]. One study reported an ORADE prevalence rate of 9.1% in previously opioid-free surgical patients [19]. In this manuscript, we report on the development of and user feedback for SPINEL (Supporting Pharmacovigilance by Leveraging Artificial Intelligence Methods to Analyze Electronic Health Records Data), a novel AI-enabled software prototype that analyzes unstructured text in discharge summaries to extract candidate ADEs for opioid drugs. FDA participants provide feedback on the serviceability of the prototype in meeting their needs to support drug safety, research, and regulatory decision-making.

## Methods

### Ethical Considerations

This study does not meet the requirements of research involving human subjects as defined by the US Department of Health and Human Services (45CFR46) for the following reasons: (1) there was no interaction or intervention with human subjects; (2) MIMIC is a free, publicly available database and the authors have completed the required Collaborative Institutional Training Initiative training and data use agreement; (3) all MIMIC III data were deidentified in accordance with Health Insurance Portability and Accountability Act requirements, including removal of 18 identifying data elements; (4) protected health information has been removed from free text fields; and (5) no personally identifiable information was available to the study investigators.

### Data Source

#### EHR Data

We limited our work to publicly accessible EHR databases and focused on the free text in discharge summaries from the Medical Information Mart for Intensive Care III (MIMIC III) [20]. This database contains EHRs from 2001 through 2012 from a single health care center; the records are encoded with codes in the International Classification of Diseases, Ninth Revision (ICD-9). We leveraged ICD-9 code E935.2, which indicates opioids and other narcotics causing AEs in therapeutic use, to prescreen discharge summaries that may contain information on ORADEs. We identified 227 summaries consisting of 227 unique hospital-event records for 226 unique patients. We planned to explore the more recently released MIMIC IV EHR database for additional cases, but the discharge summaries were not made publicly accessible until after this project was completed.

#### Reference Data Set for Testing and Training

Considering that ICD-9 codes have limited positive predictive value for drug safety surveillance [21], 2 medical students (AV, KZ) and a physician (AS) conducted independent manual reviews of the 227 discharge summaries identified by ICD-9 prescreening (as above) to manually assess for documentation of ORADEs in the text. We did not use a formal annotation guideline; positive assessments were based on specific textual mentions describing opioid drug exposure and adverse events either linked or potentially linked to the exposure irrespective of the severity or seriousness of the events. To create a reference data set of discharge summaries with true positive and negative cases, positive assessment for an ORADE required agreement among all 3 reviewers. Discrepancies were reconciled through joint discussion. The 3 reviewers had similar assessments for ORADE documentation for 174 (77%) of the 227 discharge summaries reviewed. We trained our AI-enabled model on 181 (80%) of the discharge summaries and used the remaining 46 (20%) for testing.

### NLP Process

#### Detection of Sections in Discharge Summaries

**Figure 1:**
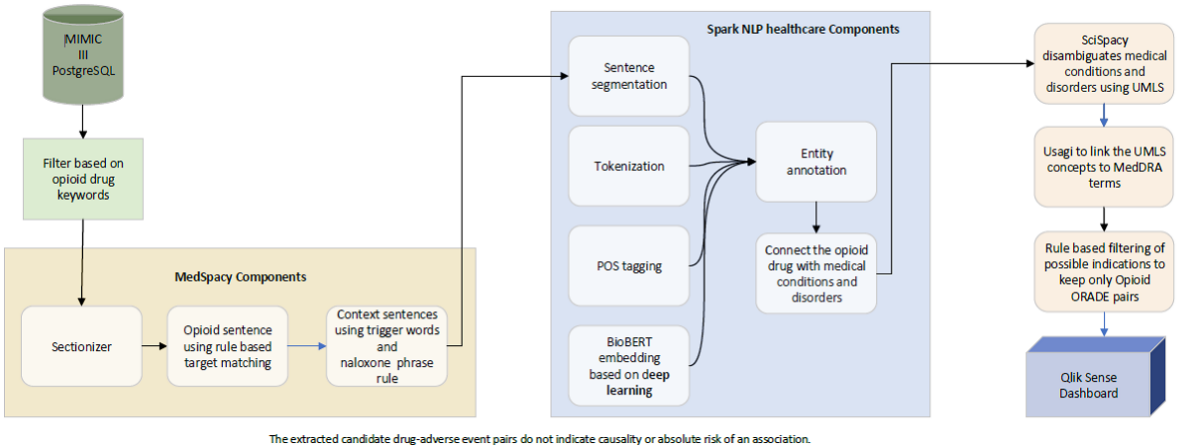
The artificial intelligence–enabled model is depicted with natural language processing and rule-based algorithms, MedSpacy sectionizer components, Spark NLP for Healthcare entity recognition components, SciSpacy disambiguation of terms, Usagi interconnection of UMLS concepts with MedDRA terminology, and further filtering of ORADE pairs. A higher resolution version of this figure is available in Multimedia Appendix 1. MIMIC: Medical Information Mart for Intensive Care; NLP: natural language processing; POS: part of speech; UMLS: Unified Medical Language System; MedDRA: Medical Dictionary for Regulatory Activities; ORADE: opioid-related adverse drug event.

Based on a manual review, we identified 3 sections with the highest frequency of ORADE mentions: “brief hospital course,” “hospital course,” and “history of present illness.” In our AI-enabled model (Figure 1), we used the Sectionizer module in the MedSpacy open-source Python library [22] to automate the identification of those component sections in the sample of discharge summaries.

#### Identifying ORADE Context Sentences Using Keywords, Trigger Phrases, and Rule-Based Algorithms

Using MedSpacy components, we divided the unstructured text in the 3 component sections of the discharge summaries into individual sentences. We identified the context sentences in 2 stages. In the first stage, we identified the sentences that contained one or more mentions of opioid-drug generic terms or opioid-drug brand names using keyword lists manually constructed by one of the team members (AS). The drug names were aligned with RxNorm terminology.

In the second stage, we used 2 rule-based approaches to identify context sentences with mentions of possible ORADEs. First, the trigger-phrase rule: We applied trigger phrases [23] to link mentions of an opioid drug with ADE terms using the MedSpacy context algorithm [24]. We curated 58 additional trigger phrases (Multimedia Appendix 2) from the training subset of the reference data set and included them in our analysis. To capture mentions of opioid drugs and ADEs that did not co-occur in the same sentence, we searched for those terms in the 3 sentences preceding and following the sentence of interest based on reported heuristics [23].

An example of a trigger-phrase rule is as follows: “It is noteworthy that the patient had received 0.5 mg Ativan x2 and morphine earlier in the afternoon and there is a concern that this may have contributed to his altered mental status.” In this context sentence, an opioid drug (“morphine”) is identified alongside a trigger phrase (“contributed to”). The Spark-NLP NER model identified the AE term as *altered mental status*. This term was resolved to the Medical Dictionary for Regulatory Activities (MedDRA) term *mental state abnormal* using Usagi (Observational Health Data Sciences and Informatics) and the corresponding UMLS concept, as in the section on disambiguation of the ORADEs below. The candidate ORADE pair generated from this information is *morphine-mental state abnormal*.

Second, the antidote-based ADE detection rule: We identified ORADE context sentences by identifying mentions of the drug naloxone, an FDA-approved medication that reverses an overdose caused by an opioid drug. To capture mentions of naloxone and opioid drugs that did not co-occur in the same sentence, we searched through the preceding and following 3 sentences. Antidote signals have been used in detecting ADRs in published literature reports [25,26].

An example of an antidote-based ADE detection rule is as follows: “He received dilaudid q 2 hr at 7:30 am, 9:30 am, 11:30 am. Code blue was called for respiratory arrest (unwitnessed). 0.4 mg of Narcan IV was administered followed by 1 mg of IV Narcan. This resulted in improvement of his respiratory status and regain of his consciousness.” In this example, the antidote-based detection rule captures mentions of naloxone in the context sentence, respiratory arrest in the preceding sentence, and the Dilaudid mention in the prior sentence to generate the candidate ORADE pair *Dilaudid-respiratory arrest.*

#### NER to Identify ORADEs in Clinical Text

Having detected opioid drug terms, we used a pretrained NER model, Spark NLP for Healthcare, which uses deep learning–based NER to identify possible AE terms in sentences. The model is a biLSTM, convolutional neural network, character–based deep learning model trained using biomedical NER data sets such as AnatEM, BC5CDR, BC4CHEMD, BioNLP13CG, JNLPBA, Linnaeus, NCBI-Disease, and S800 [27]. After identifying the AE terms in the context sentences, we connected all opioid mentions in the context sentences with the AE terms to create candidate ORADE pairs.

#### Disambiguation of ORADEs

AE terms can appear with different spellings, spelling errors, or abbreviations; therefore, we used the UMLS to map the free text to standardized concepts. We used ScispaCy to map the raw phrase found in the discharge summary to the standard UMLS translation of the concept [28]. Furthermore, we used Usagi to obtain the MedDRA term for the UMLS concept. The identified MedDRA AE term is mapped to the opioid drug term to create a candidate ORADE pair that incorporates standardized MedDRA terminology, including preferred terms (PTs) or lower-level terms (LLTs).

#### Prototype User Testing and Feedback From Participants

We recruited 15 CDER staff members to assess the various features, functionalities, and graphic visualizations. They were experienced in the use of web-based software tools but were not involved in the development of this prototype.

#### Testing Design and Conduct

A testing guide was provided that included login instructions, descriptions and screenshots of the application features and components, and instructions for exporting outputs. Test participants worked remotely, were not monitored, and were given 1 week to complete their testing. Participants were free to explore the application for their regulatory work.

For user testing, we extracted from the MIMIC III database a subset of discharge summaries filtered for an opioid drug keyword. The subset included 31,052 notes corresponding to 30,326 hospital admission events for 24,539 patients.

#### Metrics

Each participant completed an anonymous electronic survey covering technical operation, ease of navigating and interpreting various visualizations, and user satisfaction for drug safety and research (Multimedia Appendix 3).

## Results

### ORADE Detection

The prototype application successfully detected ORADEs that correspond to known opioid drug toxicities. The most commonly identified opioid drugs and the top 3 most frequent ORADEs per drug are summarized in Table 1.

To assess the contribution of keywords with trigger phrases and antidote (naloxone) signals for ORADE detection, we examined quantitative parameters for a filtered MIMIC III data subset, as shown in Table 2.

Table 2 shows that keywords with trigger phrases detect most unique AEs and candidate ORADEs in context discharge summaries. In comparison, the approach based on the antidote (ie, naloxone) makes a much smaller relative contribution to ORADE detection.

**Table 1:** Opioid-related adverse drug event detection from the text of the electronic health record discharge summaries.

Opioid drug class	Most frequently identified opioid drug	Top 3 most frequently identified opioid-related adverse drug events
Natural	Morphine	Hypotension; somnolence; nausea
Semisynthetic	Hydromorphone	Confusion; hypotension; agitation
Synthetic	Fentanyl	Hypotension; adverse reaction; hepatitis C

**Table 2:** Relative contribution of keywords with trigger phrase and antidote (ie, naloxone) signals for candidate opioid-related adverse drug event detection. International Classification of Diseases (Ninth Revision) code E935.2, which specifies opioids and other narcotics causing adverse effects in therapeutic use, was used to create a filtered subset of Medical Information Mart for Intensive Care III (MIMIC III) discharge summaries having at least one opioid-related adverse drug event pair.

	ORADE^a^ detection based on keywords with trigger phrases	ORADE detection based only on antidote (naloxone) signals	ORADE detection based on both trigger phrases and antidote signals
Number of unique opioid drugs detected (n=12)	12 (100%)	6 (50%)	6 (50%)
Number of unique AEs^b^ detected (n=117)	110 (94%)	8 (7%)	15 (13%)
Number of unique candidate ORADE pairs (n=219)	205 (94%)	13 (6%)	17 (8%)
Number of discharge summaries (n=101)	95 (94%)	8 (8%)	12 (12%)
Number of unique patients (n=101)	94 (94%)	8 (8%)	12 (12%)

^a^ORADE: opioid-related adverse drug event.

^b^AE: adverse event.

### Error Analysis

An error analysis was performed to characterize incorrect candidate ORADE pairs and is summarized with mitigation strategies in Table 3.

**Table 3:** Error analysis of false-positive and false-negative candidate opioid-related adverse drug event pairs.

Category/type and relative frequency		Example	Mitigation strategy
**False positive**			
	Drug indication pairs^a^	Text: “She was given fentanyl for the back pain with subsequent hypotension.” Incorrect candidate ORADE^b^ pair: *fentanyl-back pain*	Condition terms that include “pain” are excluded.
	Drug/medication change events^c^	Text: “She was changed from Percocet to Ultram due to nausea, which resolved.” Incorrect candidate ORADE pair: *Ultram-nausea*	The context sentence is scanned for the following phrases using regular expressions: “change to,” “switch to,” “change from drug X to drug Y,” or “switch from drug X to drug Y.” Candidate opioid drug-drug medication change event pairs so generated are excluded.
	Negated ADE^d^ mentions where the AE^e^ is not due to a drug^f^	Text: “No further apneic events.” Incorrect candidate ADE: *apneic events*	The assertion module in Spark NLP^g^ for Healthcare is used to detect negation so that any negated condition term is not included in a candidate ORADE pair.
**False negative**			
	Concept fragmentation^c^	Text: “She had been treated with high dose fentanyl and benzodiazepines which were the most likely cause of delirium.... She was also found to be severely constipated. # Constipation: patient developed severe constipation related to pain medication. She was manually disimpacted and started on an aggressive [sic] bowel regimen.” Missed candidate ORADE pair: *opioid drug-constipation*	*Severe constipation* was detected, but the current model could not find which pain medication it was related to. To resolve, we will explore more data and consider other rules or models.
	Entity not recognized as an AE	Text: “He does endorse decreased sleep latency, falling asleep in less than 5 minutes, and also questionable daytime hypersomnolence, but denies morning headaches. Of note, patient received prescription for Vicodin upon discharge from ED on [**2173-8-28**].” Missed candidate AE: *hypersomnolence*	To resolve, we will explore more data and consider other rules or models.
	Entity not recognized as an opioid drug^c^	Text: “His hospital course was complicated by a respiratory code on the floor attributed to respiratory suppression from narcotics.” Missed candidate drug: *narcotics*	*Narcotics* could be added to the opioid keyword list. To resolve to a specific opioid drug, we will explore more data and consider other rules or models.

^a^Most commonly encountered error.

^b^ORADE: opioid-related adverse drug event.

^c^Moderately encountered error.

^d^ADE: adverse drug event.

^e^AE: adverse event.

^f^Rarely encountered error.

^g^NLP: natural language processing.

### Prototype Application Performance Metrics

We calculated the performance metrics accuracy, recall, precision, and *F*_1_-score using conventional mathematical formulas [14]. The AI-enabled model achieved accuracy, recall, precision, and *F*_1_-scores of 0.66, 0.69, 0.64, and 0.67, respectively, based on the test subset of 46 discharge summaries. Candidate ORADE pairs generated with this software prototype are hypothetical and do not indicate causality or absolute risk for an association. Further assessment is required by subject matter experts.

### Prototype Application Analytics Dashboard

**Figure 2:**
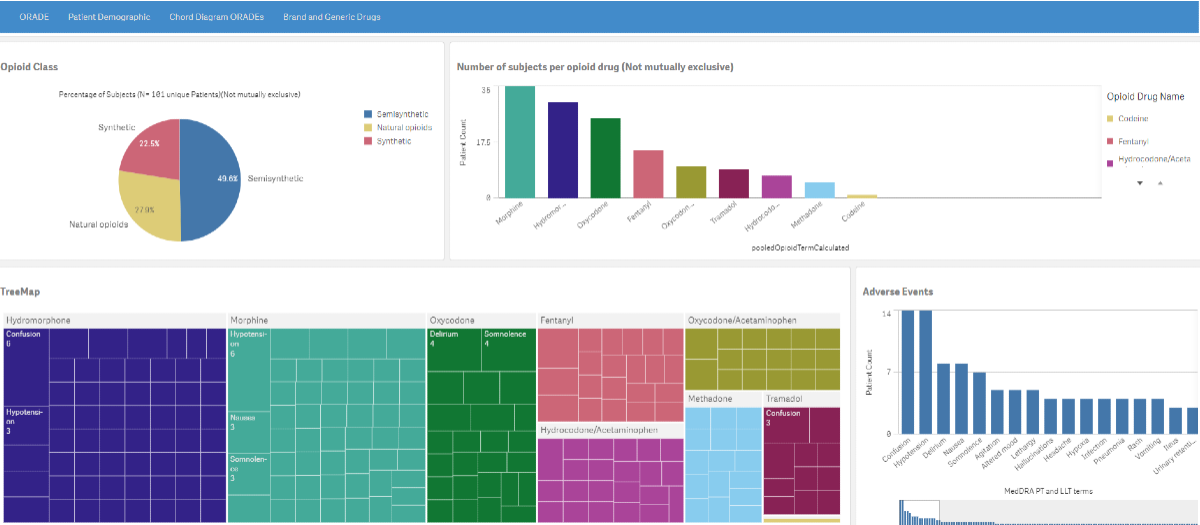
The landing page for SPINEL (Supporting Pharmacovigilance by Leveraging Artificial Intelligence Methods to Analyze Electronic Health Records Data) depicting a pie chart (upper left) of the 3 opioid classes; a histogram (upper right) of the subject counts per opioid drug; a tree map (lower left) of the electronic health record–derived opioid-related adverse drug profiles, where the adverse events for each opioid drug are represented by nested rectangles and the size of the nested rectangle relates to the patient count per adverse event; and a histogram (lower right) of patient count by MedDRA (Medical Dictionary for Regulatory Activities) preferred term and lower-level term for the drugs. A higher resolution version of this figure is available in Multimedia Appendix 1.

The Qlik Sense data analytics platform (QlikTech International AB) was used to implement the SPINEL dashboard with interactive graphics, visualizations, and line listings. The landing page (Figure 2) has 4 sheet tabs: ORADE, Patient Demographic, Chord Diagram ORADEs, and Brand and Generic Drugs. They are described below with morphine used as an arbitrarily selected opioid drug for the graphics and visualizations.

**Figure 3:**
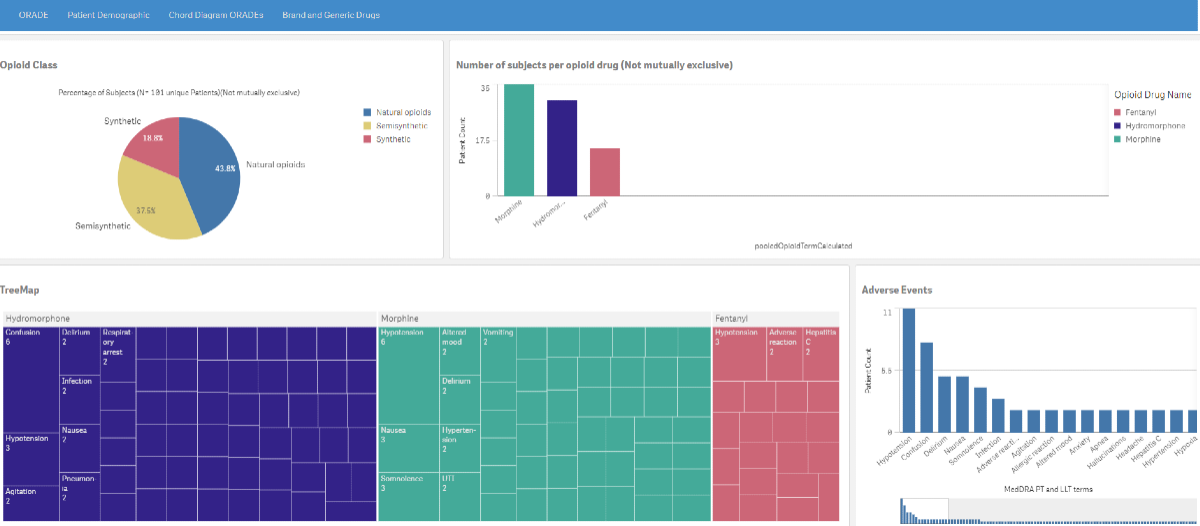
The opioid-related adverse drug page depicting a pie chart (upper left) and a histogram (upper right) of the 101 subjects who received at least one opioid class drug, a tree map (lower left) of the electronic health record–derived opioid-related adverse drug profile for the most frequently identified opioid class drugs, and a histogram (lower right) of patient count by MedDRA (Medical Dictionary for Regulatory Activities) preferred term and lower-level term for the top 3 most frequently identified opioid-related adverse drugs. A higher resolution version of this figure is available in Multimedia Appendix 1.

The ORADE tab (Figure 3) has four components: (1) a pie chart that shows subsets of the 3 classes of opioid drugs, (2) a histogram of all subjects per drug, (3) a tree map of the MedDRA PTs and LLTs for each drug, and (4) a second histogram of patient count by MedDRA PT and LLT for the selected drug(s) of interest.

**Figure 4:**
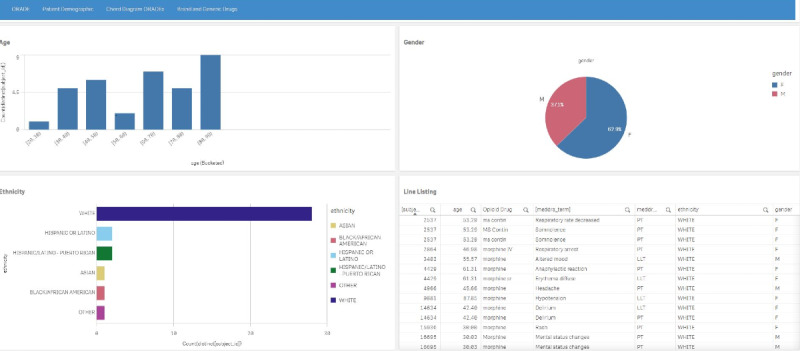
Patient demographics page depicting a histogram for morphine treated-patients by age (upper left), a pie chart for gender (upper right), a histogram for ethnicity (lower left), and a line listing (lower right) of the individual patients with adverse events and associated demographics. Morphine is an arbitrarily selected natural opioid drug. A higher resolution version of this figure is available in Multimedia Appendix 1.

The patient demographic tab (Figure 4) includes the following components: (1) a histogram of age, (2) a pie chart of gender, (3) another histogram of ethnicity, and (4) a line listing of the individual patients with AEs and associated demographics.

**Figure 5:**
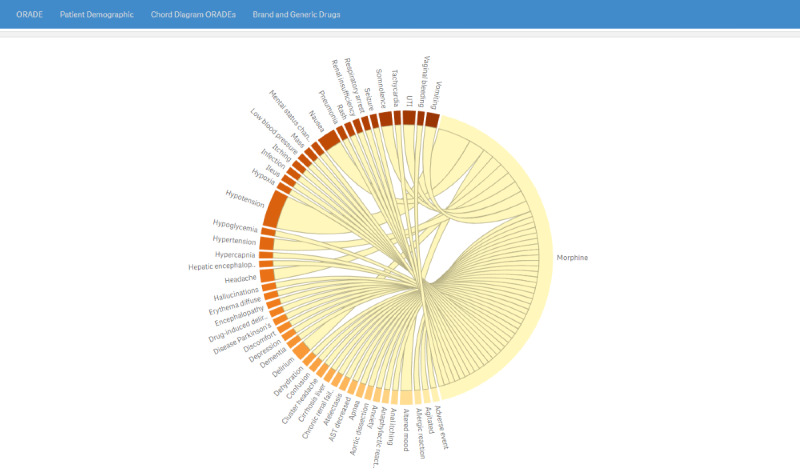
Cord diagram page visually depicting the interconnections between the opioid drug of interest (morphine in this example) and adverse event mentions as derived from the electronic health record discharge summaries. The larger the caliber of the connecting cord, the higher the adverse drug event frequency. Morphine is an arbitrarily selected natural opioid drug. A higher resolution version of this figure is available in Multimedia Appendix 1.

The chord diagram tab (Figure 5) displays a graphic to visually explore interconnections between opioid drugs and AE mentions.

**Figure 6:**
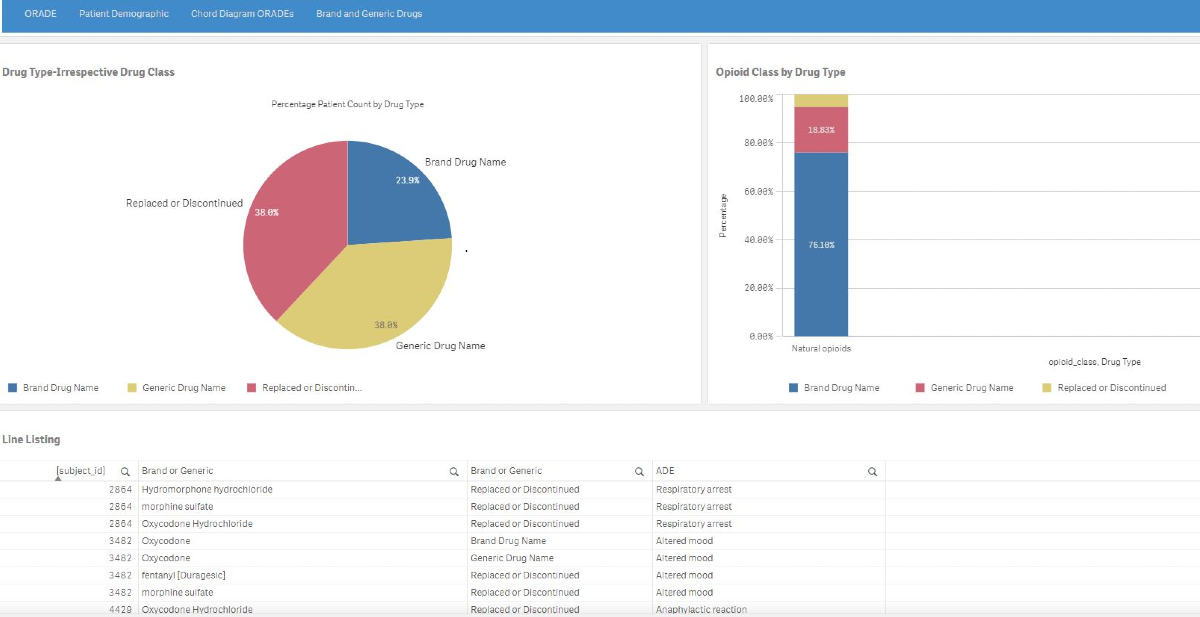
Brand and generic drugs page depicting a pie chart (upper left) of brand, generic, or replaced/discontinued drug type, a stacked bar chart (upper right) of patients by type, and a line listing (lower section) of the patients by drug name, drug type, and adverse events. A higher resolution version of this figure is available in Multimedia Appendix 1.

The brand and generic drugs tab (Figure 6) includes multiple displays: (1) a pie chart with the percentage patient count by brand or generic drug type, (2) a stacked bar chart of patients by opioid class and drug type, and (3) a searchable, scrollable spreadsheet listing of the drug name, drug type, and adverse events associated with the subject IDs.

### Results of User Testing

SPINEL was assessed as a highly desirable prototype that satisfies end user needs for supporting opioid drug safety signal detection from EHR data. The application was easy to use, the visualizations enhanced detection of drug safety signals, and the prototype ranked high in saving time compared to manual chart review. Survey results were based on a Likert rating scale (Multimedia Appendix 4).

Fifteen FDA staff completed the survey questionnaire with 11 providing observational feedback. Participant feedback uncovered a few minor bugs and indicated the following areas for potential improvement: (1) enlarge the tabs and visualizations, (2) enable more flexibility and customizations to fit each end user’s needs, (3) provide additional instructional resources to enhance learning about the various features and functionalities, (4) add multiple graph export functionality, and (5) add project summaries. Possible mitigation strategies include adding a slider bar with zoom function for the more complex visualizations, providing an instructional video on the application’s features and functionalities, providing tool-tip pop-ups and a supplemental “user tips” guide to highlight key features or functionality, modifying the export function to accommodate multiple graphics, and developing a customizable user portal to include project summaries.

## Discussion

### Principal Results

The AI-enabled SPINEL prototype successfully detects known opioid drug toxicities from free text in EHRs and provides a framework to uncover emerging safety data that could potentially augment regulatory review and decision-making. Automated processing and analysis of EHR data reduces the research burden compared to manual chart review, saving considerable time and effort. The prototype expedites the quick perusal of data for trends and patterns reflecting drug toxicities while facilitating drilling down into the data to patient-level line listing information. FDA participants conveyed high satisfaction ratings for this prototype and acknowledged its potential to add value in harnessing unstructured text in EHRs for pharmacovigilance.

In applying our AI-based model, we limited our analysis to discharge summaries because published studies confirm that discharge summaries are the best subsection of the EHR for gathering information about ADEs reported by physicians [29-31]. In reviewing the discharge summaries, we observed considerable heterogeneity in the quality of reporting and the depth of detail conveyed about possible ORADEs, which could affect the accuracy and other performance metrics for the software application. We applied 2 rule-based algorithms to enhance ORADE detection from discharge summaries. Our results demonstrate that the majority of candidate ORADE pairs and context discharge summaries are detected using keywords with trigger phrases. As described in published literature [32], this approach to searching for drug safety signals is best for uncovering ADEs potentially related to specific drug products as delineated in the keyword list (opioids in our use case). As new drug products are approved by the FDA, the keyword list would need manual updating to keep it current. However, for broader and more generalized searching, this could become cumbersome, as new keyword lists would need to be manually compiled for each drug grouping or class of interest.

The accuracy, recall, and precision of this prototype will need to be improved to better align with established NLP processors. Two steps to be considered in future work to improve performance are (1) leveraging information from established drug databases, such as the DailyMed database of the most recent FDA-approved drug product labels to filter out false positive ORADEs due to drug-indication pairs and (2) using large language models (LLMs) such as GPT-4 [33], BioGPT [34], or GatorTron [35] to improve capture of mentions of opioid drugs and ADE terms that may be separated by multiple paragraphs.

### Limitations

This project encountered three main challenges and limitations. First, patient cohort identification: Use of ICD-9 codes to prescreen discharge summaries for potential cases of ORADEs could be impacted by selection and misclassification biases resulting in a subset that may not reflect the total number of ORADE cases in the MIMIC III data. These biases could result in a skewed patient sample wherein there may be missed patients with ORADEs or patients incorrectly classified as having an ORADE due to erroneous coding. In addition, in focusing only on the free-text discharge summaries, we may have missed patients whose ORADEs were captured only in other text reports that we did not explore, such as physician notes, nursing notes, and consultation reports. Together, these issues may prevent us from capturing the full extent and scope of patients experiencing ORADEs from the MIMIC III EHRs. In future work, a more robust approach to identifying patients with ORADEs will be considered, including use of a standardized annotation guideline and reporting of interannotator agreement scores related to development of a reference data set; possible inclusion of objective components for case ascertainment, such as laboratory or medical imaging abnormalities; and expanding the scope of reports assessed to include physician notes, nursing notes, and consultation reports, where available, in addition to discharge summaries. Second was the use of MIMIC III. The single-center MIMIC III EHR database may not reflect the broad diversity of the US population, which could limit generalizability for drug safety surveillance to larger and more diversified domains and lend to potentially biased assessments. Third, the lack of a publicly available reference standard data set hindered efforts to evaluate the NLP component of our AI-enabled model in detecting ADE safety signals from text in EHRs. The small size of our reference data set risked overfitting and biased assessments.

There were limitations inherent in the user testing procedures. User testing was unmonitored and conducted without prespecified tasks. This approach accommodated participants working in remote locations to explore the software in their regulatory work. However, direct observation by a facilitator may have enabled us to gather more details about end-user experience. Additionally, the sample size of intended users was small. Feedback from a larger group of CDER regulatory staff may be more informative about the potential impact on their regulatory work and decision-making.

### Conclusions

SPINEL, our novel AI-enabled software, extracts ORADEs from free-text discharge summaries in EHRs, streamlines workflow, and augments access to real world data for pharmacovigilance. Detecting opioid safety signals from EHRs enhances the capacity to harness an important yet underutilized resource of clinically relevant information for regulatory review and decision-making.

Future work will explore detecting newly emerging opioid drug safety issues using a larger and more diversified EHR database, investigating various methods to improve NLP performance, resolving application features per FDA participant feedback, and integrating knowledge graphs to interconnect information from EHRs with reports published in the literature.

## Appendix

Multimedia Appendix 1 High resolution versions of Figures 1-6.

Multimedia Appendix 2 List of 58 newly curated trigger phrases.

Multimedia Appendix 3 Anonymous user survey questions.

Multimedia Appendix 4 User Survey: Likert scale ratings from user testing.

## Abbreviations

ADE: adverse drug event
ADR: adverse drug reaction
AE: adverse event
AI: artificial intelligence
BERT: bidirectional encoder representations from transformers
CDER: Center for Drug Evaluation and Research
CNN: convolutional neural network
CRF: conditional random field
cTAKES: Clinical Text Analysis and Knowledge Extraction System
EHR: electronic health record
FAERS: FDA Adverse Events Reporting System
FDA: US Food and Drug Administration
GPT: generative pretrained transformer
ICD-9: International Classification of Diseases, Ninth Revision
LLM: large language model
LLT: lower-level term
LSTM: long short-term memory
MedDRA: Medical Dictionary for Regulatory Activities
MIMIC: Medical Information Mart for Intensive Care
NER: named entity recognition
NLP: natural language processing
ORADE: opioid-related adverse drug event
POS: part of speech
PT: preferred term
SPINEL: Supporting Pharmacovigilance by Leveraging Artificial Intelligence Methods to Analyze Electronic Health Records Data
SVM: support vector machine
UMLS: Unified Medical Language System
