# Supplementary material for: Artificial Intelligence–Enabled Software Prototype to Inform Opioid Pharmacovigilance From Electronic Health Records: Development and Usability Study
Source: JMIR AI. 2023 Jul 18;2:e45000. doi: 10.2196/45000 (PMC10538589; doi:10.2196/45000)
Supplement: Multimedia Appendix 1 [file ai_v2i1e45000_app1.pdf]

Figure S1 The artificial intelligence–enabled model is depicted with natural language processing and rule-based algorithms, MedSpacy sectionizer components, Spark NLP for Healthcare entity recognition components, SciSpacy disambiguation of terms, Usagi interconnection of UMLS concepts with MedDRA terminology, and further filtering of ORADE pairs. MIMIC: Medical Information Mart for Intensive Care; NLP: natural language processing; POS: part of speech; UMLS: Unified Medical Language System; MedDRA: Medical Dictionary for Regulatory Activities; ORADE: opioid-related adverse drug event.

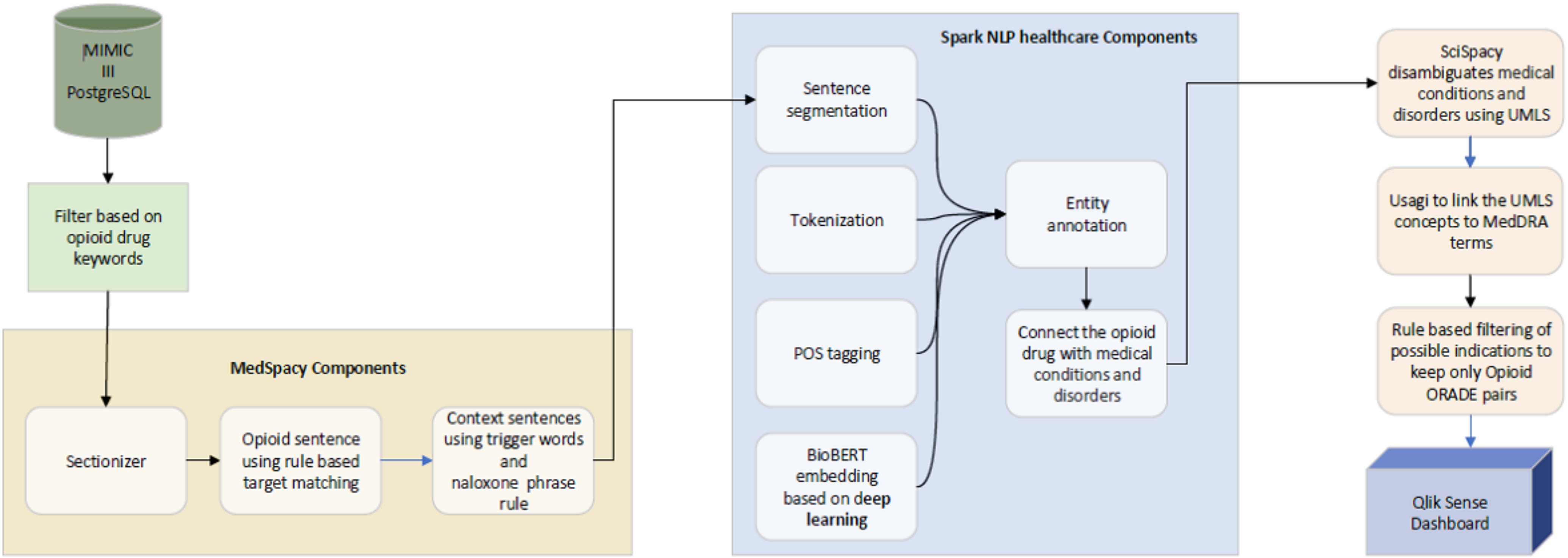

The extracted candidate drug-adverse event pairs do not indicate causality or absolute risk of an association.

Figure S2

The landing page for SPINEL (Supporting Pharmacovigilance by Leveraging Artificial Intelligence Methods to Analyze Electronic Health Records Data) depicting a pie chart (upper left) of the 3 opioid classes; a histogram (upper right) of the subject counts per opioid drug; a tree map (lower left) of the electronic health record–derived opioid-related adverse drug profiles, where the adverse events for each opioid drug are represented by nested rectangles and the size of the nested rectangle relates to the patient count per adverse event; and a histogram (lower right) of patient count by MedDRA (Medical Dictionary for Regulatory Activities) preferred term and lower-level term for the drugs.

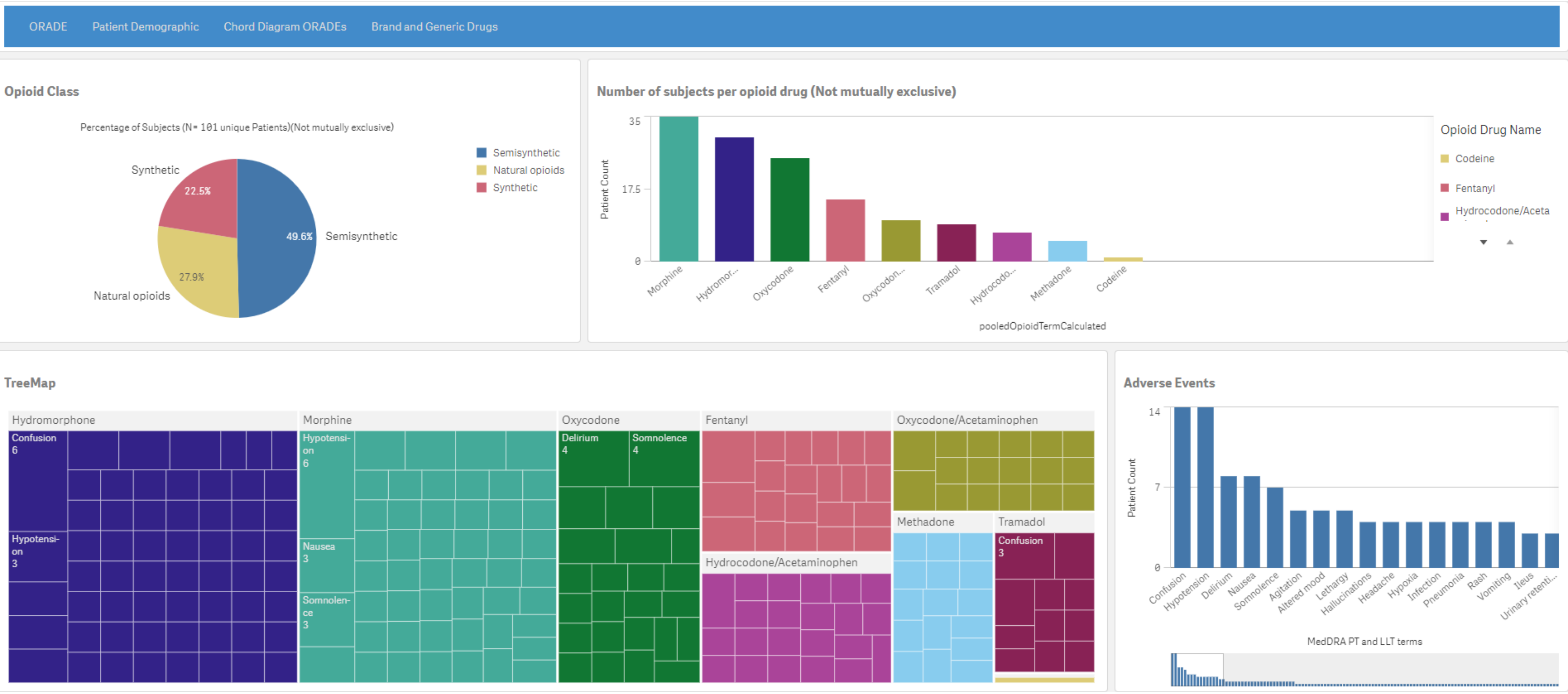

Figure S3 The opioid-related adverse drug page depicting a pie chart (upper left) and a histogram (upper right) of the 101 subjects who received at least one opioid class drug, a tree map (lower left) of the electronic health record–derived opioid-related adverse drug profile for the most frequently identified opioid class drugs, and a histogram (lower right) of patient count by MedDRA (Medical Dictionary for Regulatory Activities) preferred term and lower-level term for the top 3 most frequently identified opioid-related adverse drugs.

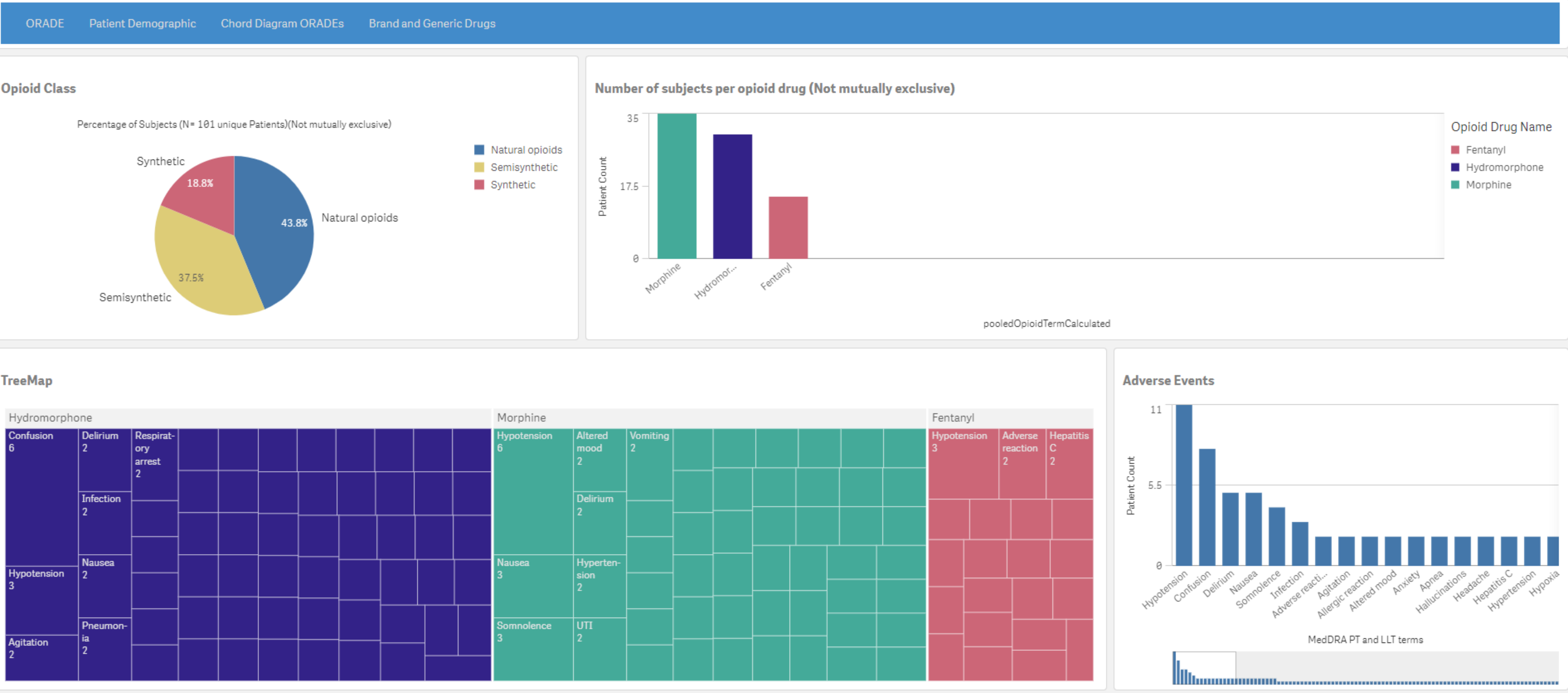

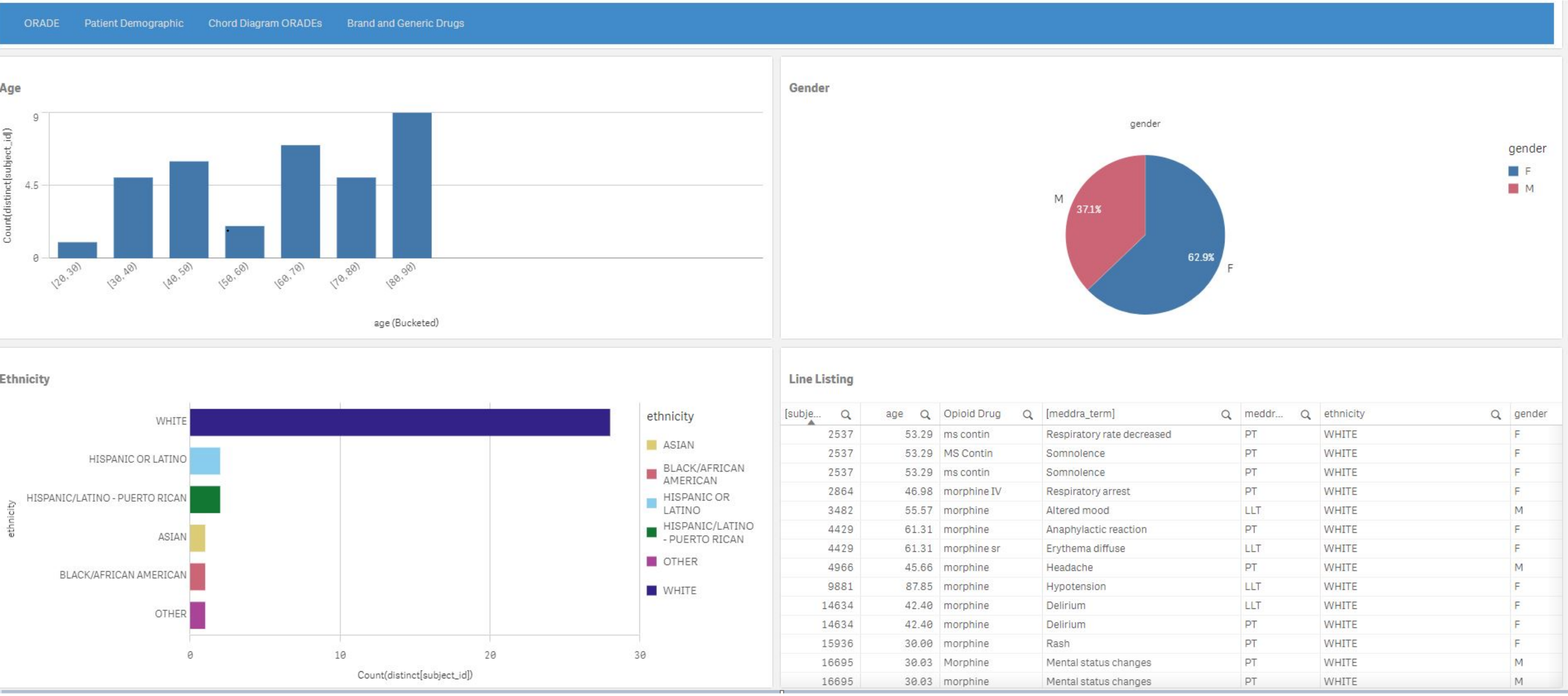

Figure S4 Patient demographics page depicting a histogram for morphine treated-patients by age (upper left), a pie chart for gender (upper right), a histogram for ethnicity (lower left), and a line listing (lower right) of the individual patients with adverse events and associated demographics. Morphine is an arbitrarily selected natural opioid drug.

Figure S5

Cord diagram page visually depicting the interconnections between the opioid drug of interest (morphine in this example) and adverse event mentions as derived from the electronic health record discharge summaries. The larger the caliber of the connecting cord, the higher the adverse drug event frequency. Morphine is an arbitrarily selected natural opioid drug.

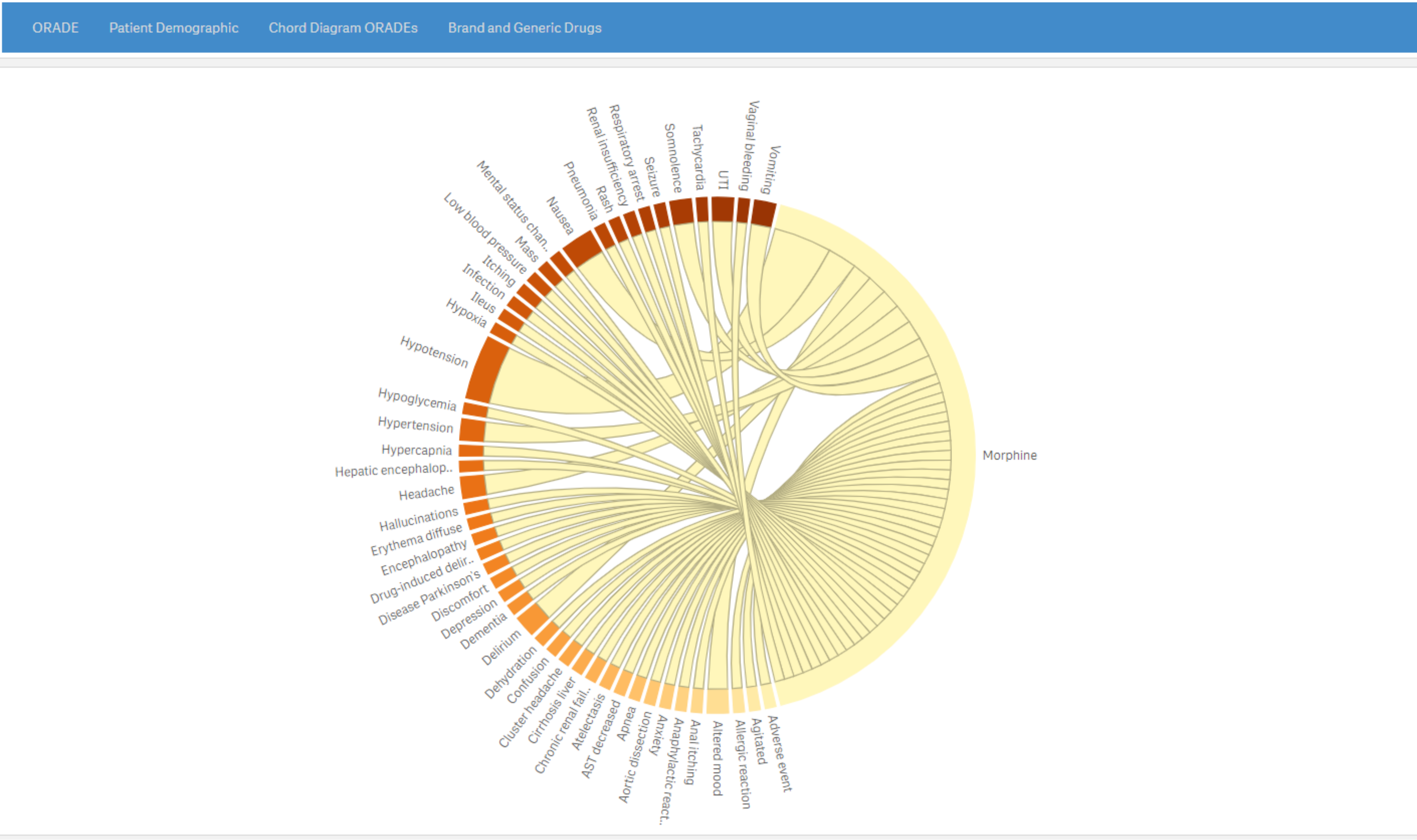

Figure S6 Brand and generic drugs page depicting a pie chart (upper left) of brand, generic, or replaced/discontinued drug type, a stacked bar chart (upper right) of patients by type, and a line listing (lower section) of the patients by drug name, drug type, and adverse events.

ORADE | Patient Demographic | Chord Diagram ORADEs | Brand and Generic Drugs

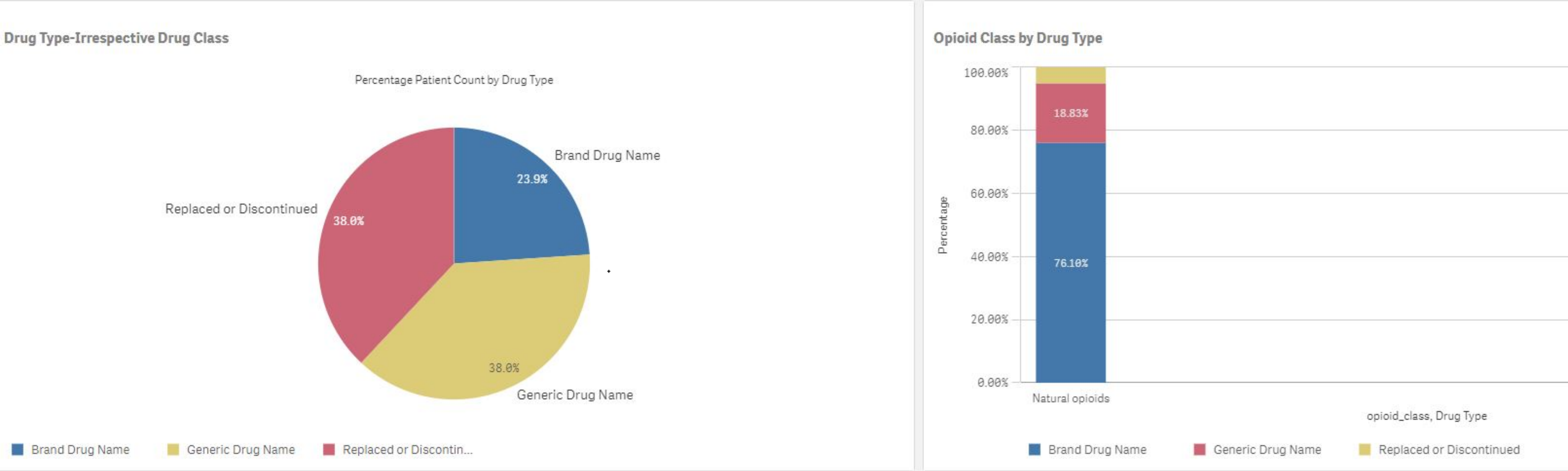

Line Listing

[subject_id]	Brand or Generic	Brand or Generic	ADE
2864	Hydromorphone hydrochloride	Replaced or Discontinued	Respiratory arrest
2864	morphine sulfate	Replaced or Discontinued	Respiratory arrest
2864	Oxycodone Hydrochloride	Replaced or Discontinued	Respiratory arrest
3482	Oxycodone	Brand Drug Name	Altered mood
3482	Oxycodone	Generic Drug Name	Altered mood
3482	fentanyl [Duragesic]	Replaced or Discontinued	Altered mood
3482	morphine sulfate	Replaced or Discontinued	Altered mood
4429	Oxycodone Hydrochloride	Replaced or Discontinued	Anaphylactic reaction
