# Supplementary material for: Artificial Intelligence–Enabled Software Prototype to Inform Opioid Pharmacovigilance From Electronic Health Records: Development and Usability Study
Source: JMIR AI. 2023 Jul 18;2:e45000. doi: 10.2196/45000 (PMC10538589; doi:10.2196/45000)
Supplement: Multimedia Appendix 2 [file ai_v2i1e45000_app2.pdf]

Appendix 1

**Trigger phrase**	**Count***
made	223
decreased	209
resolved	167
noted to	98
felt	79
related to	63
concern for	49
episodes of	48
episode of	40
code	36
likely secondary to	35
likely due to	33
subsequent	29
administration of	28
episodes	27
complicated by	26
episode	23
worsened	23
most likely	21
causing	20
improved after	19
resulted in	19
thought to be	19
contributed to	16
contributing to	16
sensitive to	15
switched from	13
reaction to	12
changed from	11
exacerbated by	11
likely from	11
reaction	10
discontinued due to	9
possibility	9
felt to	9
cause of	9
unresponsive after	7
felt to be	7
likely related to	6

allergic reaction to	6
changed back to	5
triggered	4
medication effect	4
most likely secondary to	4
discontinuing	4
change from	3
resolved upon	3
thought to be from	3
believed to be related to	2
down titrate	1
presumed to be	1
discontinued	1
reversed with	1
concomitantly	1
determined to be from	1
multifactorial due to	1
side effect of	1
felt most likely	1

*Count of trigger phrases that were associated with at least one ORADE in one context sentence
