# Supplementary material for: Artificial Intelligence–Enabled Software Prototype to Inform Opioid Pharmacovigilance From Electronic Health Records: Development and Usability Study
Source: JMIR AI. 2023 Jul 18;2:e45000. doi: 10.2196/45000 (PMC10538589; doi:10.2196/45000)
Supplement: Multimedia Appendix 3 [file ai_v2i1e45000_app3.pdf]

## Appendix 2

1. Please indicate your FDA affiliation.

2. The following statements are to be rated (Strongly Disagree, Disagree, Neutral, Agree, or Strongly Agree)

    1. SPINEL is easy to learn and operate
    2. The SPINEL dashboard has a common-sense layout
    3. The data visualizations are easy to understand and interpret
    4. The dashboard responds rapidly to changes in the search query
    5. SPINEL supports Web browser compatibility other than Google Chrome
    6. SPINEL's visualizations help me to quickly identify trends and patterns in opioid drug safety signals from electronic health records narratives
    7. SPINEL saves time compared to manual health record chart review
    8. The SPINEL user interface meets my needs
    9. I am satisfied with my experience using SPINEL
    10. I would recommend SPINEL to a colleague

3. Do you have any feedback on SPINEL?
