# Supplementary material for: Artificial Intelligence–Enabled Software Prototype to Inform Opioid Pharmacovigilance From Electronic Health Records: Development and Usability Study
Source: JMIR AI. 2023 Jul 18;2:e45000. doi: 10.2196/45000 (PMC10538589; doi:10.2196/45000)
Supplement: Multimedia Appendix 4 [file ai_v2i1e45000_app4.pdf]

Multimedia Appendix 3

	STRONGLY DISAGREE n (%)	DISAGREE n (%)	NEUTRAL n (%)	AGREE n (%)	STRONGLY AGREE n (%)
SPINEL is easy to learn and operate	0 (0%)	0 (0%)	0 (0)	6 (40%)	9 (60%)
The SPINEL dashboard has a common-sense layout	0 (0%)	0 (0%)	0 (0)	5 (33%)	10 (67%)
The data visualizations are easy to understand and interpret	0 (0%)	0 (0%)	1 (7%)	3 (20%)	11 (73%)
The dashboard responds rapidly to changes in the search query	0 (0%)	0 (0%)	0 (0)	4 (27%)	11 (73%)
SPINEL supports Web browser compatibility other than Google Chrome	0 (0%)	0 (0%)	3 (20%)	1 (7)%	11 (73%)
SPINEL's visualizations help me to quickly identify trends and patterns in opioid drug safety signals from electronic health records narratives	0 (0%)	0 (0%)	1 (7%)	3 (20%)	11 (73%)
SPINEL saves time compared to manual health record chart review	0 (0%)	0 (0%)	0 (0%)	1 (7%)	13 (93%)
The SPINEL user interface meets my needs	0 (0%)	0 (0%)	1 (7%)	3 (20%)	11 (73%)
I am satisfied with my experience using SPINEL	0 (0%)	0 (0%)	0 (0%)	4 (27%)	11 (73%)
I would recommend SPINEL to a colleague	0 (0%)	0 (0%)	0 (0%)	4 (27%)	11 (73%)

User Survey: Likert scale ratings
